# SCALOP: sequence-based antibody canonical loop structure annotation

**DOI:** 10.1093/bioinformatics/bty877

**Published:** 2018-10-15

**Authors:** Wing Ki Wong, Guy Georges, Francesca Ros, Sebastian Kelm, Alan P Lewis, Bruck Taddese, Jinwoo Leem, Charlotte M Deane

**Affiliations:** 1 Department of Statistics, University of Oxford, Oxford, UK; 2 Roche Pharma Research and Early Development, Large Molecule Research Roche Innovation Center Munich, Penzberg, Germany; 3 UCB Pharma, Slough, UK; 4 Computational and Modelling Sciences, GlaxoSmithKline Research and Development, Stevenage, UK; 5 Antibody Discovery and Protein Engineering, MedImmune, Granta Park, Cambridge, UK

## Abstract

**Motivation:**

Canonical forms of the antibody complementarity-determining regions (CDRs) were first described in 1987 and have been redefined on multiple occasions since. The canonical forms are often used to approximate the antibody binding site shape as they can be predicted from sequence. A rapid predictor would facilitate the annotation of CDR structures in the large amounts of repertoire data now becoming available from next generation sequencing experiments.

**Results:**

SCALOP annotates CDR canonical forms for antibody sequences, supported by an auto-updating database to capture the latest cluster information. Its accuracy is comparable to that of a standard structural predictor but it is 800 times faster. The auto-updating nature of SCALOP ensures that it always attains the best possible coverage.

**Availability and implementation:**

SCALOP is available as a web application and for download under a GPLv3 license at opig.stats.ox.ac.uk/webapps/scalop.

**Supplementary information:**

Supplementary data are available at *Bioinformatics* online.

## 1 Introduction

Antibodies are proteins of the immune system that bind to foreign molecules. The binding site is largely formed of six complementarity-determining regions (CDRs): three on each of the heavy and light chains. Conformational clusters, known as ‘canonical forms’, have been observed in five of the six CDRs (e.g. Chothia and Lesk, 1987; North *et al.*, 2011; Nowak *et al.*, 2016). Canonical forms have been redefined in the literature many times, but each update has been a static snapshot of the available data. These constant renewals illustrate how the growth of structural data continuously modifies our understanding of CDR loop structures, with 10 canonical forms in 1987 (Chothia and Lesk, 1987) and 26 by 2016 (Nowak *et al.*, 2016).

Several sequence-based canonical form prediction methods have been developed (e.g. Chothia and Lesk, 1987; Long *et al.*, 2018; North *et al.*, 2011; Nowak *et al.*, 2016). Chothia and Lesk (1987) suggested structurally-determining residues for canonical form assignment. Using a similar approach, Swindells *et al.* (2017) published a freely available web server that can handle bulk canonical form assignment, but some clusters lack a representative structure. Hidden Markov models have also been built for cluster assignment (North *et al.*, 2011; Nowak *et al.*, 2016). The most recently published method used a Gradient Boosting Machine to annotate CDR backbone conformations with up to 85.1% accuracy (Long *et al.*, 2018). However, none of these tools uses an auto-updating database, and none provides both a web interface and a freely available software package for large-scale sequence analysis.

Here we present SCALOP, which both clusters the H1, H2, L1, L2 and L3 CDRs in an auto-updating database, and creates a canonical form predictor. SCALOP can be used to rapidly approximate an antibody binding site shape from sequence alone (Krawczyk *et al.*, 2018) with a minimum accuracy of 89.47% (Table 1) (Supplementary Material). The tool is available as a web server and as a Python package for bulk processing.

**Table 1.** Coverage and precision of SCALOP and FREAD on SAbDab

		H1	H2	L1	L2	L3
Coverage (%)	SCALOP	93.75	97.54	97.38	98.50	91.69
	FREAD	96.79	93.38	98.76	98.89	98.02
Precision (%)	SCALOP	89.26	93.60	95.67	99.13	93.31
	FREAD	80.19	88.50	92.72	98.27	91.29

*Note*: A target structure with a root-mean-square deviation of <1.5 Å to the predicted structure is considered correct.

## 2 Algorithm

SCALOP takes one or a set of amino acid sequences of full antibody chains as input. It then numbers the sequence with ANARCI (Dunbar and Deane, 2016), and scores the extracted CDR sequences against PSSMs of the appropriate clusters. The cluster nomenclature follows that of Nowak *et al.* (2016) (Supplementary Material). The input CDR sequence is then assigned to the cluster with the maximum score above a scoring threshold (Supplementary Material). SCALOP returns the name of the assigned cluster, and the PDB code and chain identifier of the assigned cluster’s median structure as the result. SCALOP can return a structural model if a structure of the framework is given alongside the CDR sequence (Supplementary Material). The database is updated monthly, previous databases are available from the website.

### 2.1 Building the PSSM

We adopted the length-independent CDR clustering method developed by Nowak *et al.* (2016). Structures in SAbDab (Dunbar *et al.*, 2014) available as of July 10, 2017 were used (Supplementary Material). We built PSSMs for each cluster using their unique sequences only:

$$M_{k,j}=log(\frac{p_{k,j}}{b_{k}})$$

where $M_{k,j}$ is the element score, $p_{k,j}$ is the probability of observing an amino acid k at the ANARCI-numbered position j within the cluster and $b_{k}$ is the background probability of k (Supplementary Material).

### 2.2 Cluster assignment

To make a cluster prediction, we only consider the target sequence against clusters of the respective CDR types (i.e. H1 or H2). The PSSM score for a target sequence, $s_{c}$ for cluster c is:

$$s_{c}=\sum_{j=J_{0}}^{J}M_{k,j}$$

where J is the set of positions in the target sequence. Since L2 loop structures are often invariant, we assign L2 loops of the dominant sequence length to a single canonical form; otherwise, it is not clustered.

## 3 Benchmark

We evaluated the performance of SCALOP on our training set using a leave-one-out cross-validation protocol (Table 1) and on a blind test set (Supplementary Material). It achieved similar results on both. We also compared to an adapted version of FREAD, an accurate database-search method for loop structure prediction (Deane and Blundell, 2001; Krawczyk *et al.*, 2018) (Supplementary Material). This version does not generate a structural model, but returns the PDB code of its prediction. The prediction coverage and precision of the methods are comparable (Table 1) (Supplementary Material).

To assess the speed and the portion of consistent predictions made by SCALOP and FREAD, we ran both predictors on a next generation sequencing dataset, with ∼8 million light chain and ∼5 million heavy chain sequences (Krawczyk *et al.*, 2018). About 98% of the predictions are consistent between the two methods (Supplementary Material). On a single core, predicting 100 sequences requires 227s using FREAD, but 0.29s using SCALOP. This rapid prediction suggests the possibility of running SCALOP as a fast and reliable first-screen.

**Fig. 1.**
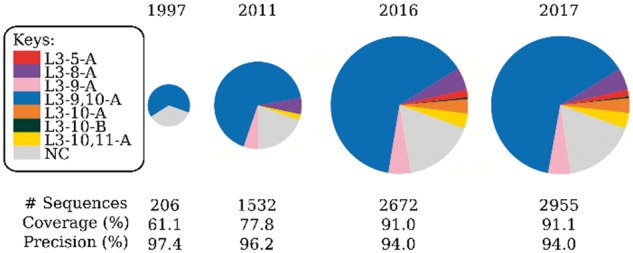
The changes in L3 clusters in the past 20 years. The radii of the pie charts are proportional to the log(number of sequences). In 1997, only one L3 cluster existed whose members were all length-9 loops. In 2011, four clusters existed, covering different sequence lengths. Between 2011 and 2016, some length-10 sequences joined the 2011-L3-9-A cluster, which becomes the 2016-L3-9, 10-A cluster. The enriched knowledge improves the prediction coverage of SCALOP while retaining the precision. The numbers below the pie chart are a leave-one-out (if needed) cross-validation on all antibodies up to July 1, 2018 (Supplementary Material)

In order to ensure that SCALOP always offers the best possible prediction coverage, it uses an auto-updating database. Figure 1 demonstrates the advantage of this auto-updating approach using L3 as an example. We selected the representative years based on previous publication dates of canonical forms definitions (Al-Lazikani *et al.*, 1997; North *et al.*, 2011; Nowak *et al.*, 2016). Data until the end of the year were used, i.e. for 2016, all structures available on SAbDab deposited before the end of 2016 were used. In 1997, there was only a single L3 cluster; by 2016 there were seven and the portion of non-clustered data had more than halved. Using the 1997 dataset for prediction, we achieve similar precision as with 2017’s data (97.4% in 1997 and 94.0% in 2017), but ∼30% less coverage.

## Funding 

This work was supported by funding from the Engineering and Physical Sciences Research Council and Medical Research Council [grant number EP/L016044/1].

*Conflict of Interest*: none declared.

## Supplementary Material

bty877_Supplementary_Materials
