# Supplementary material for: SCALOP: sequence-based antibody canonical loop structure annotation
Source: Bioinformatics. 2018 Oct 15;35(10):1774–6. doi: 10.1093/bioinformatics/bty877 (PMC6513161; doi:10.1093/bioinformatics/bty877)
Supplement: bty877_Supplementary_Materials [file bty877_supplementary_materials.pdf]

Supplementary Materials for SCALOP: sequence-based antibody canonical loop structure annotation

S1 Length-independent clustering of complementarity-determining region (CDR) structures 1

S1.1 CDR Loop extraction from the PDB 1

S1.2 Cluster formation 1

S1.3 Cluster nomenclature 1

S1.4 Summary statistics of the clusters 2

S2 Construction of the position-specific scoring matrix and scoring method 4

S3 Cross-validation for threshold selection 5

S4 Blind test set for SCALOP prediction performance 7

S5 Cross-validation for structure selection 8

S6 Benchmark with FREAD 9

S6.1 Coverage and precision of FREAD on loops in the SCALOP database 9

S7 Performance of SCALOP and FREAD on next generation sequencing (NGS) data 10

S8 Backdating the SCALOP database: Performance evaluation 14

References 17

S1 Length-independent clustering of complementarity-determining region (CDR) structures

We followed the protocol and nomenclature outlined by Nowak *et al.* (2016) to carry out length-independent clustering of the CDR structures.

S1.1 CDR Loop extraction from the PDB

All X-ray structures available in SAbDab (Dunbar *et al.* 2014) as of 10th July 2017, with a resolution of ≤ 2.8 Å, were considered in this work. For this paper, we adopted the IMGT numbering scheme (Lefranc *et al.*, 2009) and the CDR definition described by North *et al.* (2011). CDR loops with no missing residues and no B-factors of backbone atoms ≥ 80 were considered.

S1.2 Cluster formation

Five residues before and five after the CDR termini were used as the anchors for structural alignment. Pairwise backbone root-mean-square deviation (RMSD) between loop structures were calculated to form the cost matrix. For loop structures that differ in length, a dynamic time warping (DTW) algorithm was used to find the optimal structural alignment between the backbone atoms. Density-based spatial clustering of applications with noise (DBSCAN) was used to carry out the structural clustering. The clustering thresholds are the same as in Nowak *et al.* (2016), except for L2 where a clustering threshold of 1 Å was used.

S1.3 Cluster nomenclature

The cluster nomenclature follows that of Nowak *et al.* (2016). A cluster is named as follows: the first two letters represent the type of CDR (H1 or H2 *etc.*), followed by the sequence lengths found in the cluster, and completed with an alphabet representing the rank of the cluster in descending sizes. For instance, an L3 cluster which contains length-10 and length-11 sequences (10,11), and has the second highest number of unique sequences among all clusters which contain both length-10 and length-11 sequences (B), is called 'L3-10,11-B'.

S1.4 Summary statistics of the clusters

We defined a cluster as a set of CDR structures with at least 6 unique sequences.

Table S1.1 Summary statistics of clusters in each CDR type.

CDR	Total number of sequences	Clustering Threshold (Å)	Portion of clustered sequences	Number of clusters
**H1**	2747	0.80	81.03%	3
**H2**	2819	0.63	83.29%	4
**L1**	2605	0.82	92.32%	12
**L2**	2765	1	98.41%	1
**L3**	2713	0.91	83.27%	7

Table S1.2 Cluster-specific details of each CDR type.

CDR	Clusters	Lengths	#Unique	#Redundant
**H1**	H1-13-A	13	605	2047
	H1-14-A	14	20	80
	H1-15-A	15	20	99
	**#Clustered**	**2226**	**#Total**	**2747**
**H2**	H2-9-A	9	170	608
	H2-10-A	10	366	1001
	H2-10-B	10	187	561
	H2-12-A	12	39	178
	**#Clustered**	**2348**	**#Total**	**2819**
**L1**	L1-10-A	10	26	80
	L1-11-A	11	243	1051
	L1-11-B	11	40	120
	L1-12-A	12	24	54
	L1-12-B	12	11	87
	L1-13-A	13	33	95
	L1-13-B	13	9	47
	L1-13-C	13	7	20
	L1-14-A	14	21	91
	L1-14-B	14	11	100
	L1-15-A	15	40	97
	L1-16,17-A	16,17	144	563
	**#Clustered**	**2405**	**#Total**	**2605**
**L2**	L2-8-A	8	449	2721
	**#Clustered**	**2721**	**#Total**	**2765**
**L3**	L3-5-A	5	12	49
	L3-8-A	8	40	141
	L3-9-A	9	29	141
	L3-9,10-A	9,10	470	1729
	L3-10-A	10	20	92
	L3-10-B	10	8	10
	L3-10,11-A	10,11	41	97
	**#Clustered**	**2259**	**#Total**	**2713**

S2 Construction of the position-specific scoring matrix and scoring method

We constructed the position-specific scoring matrix (PSSM) based on the frequency of amino acids found in that position within the cluster:

$$M_{k,j} = log_2(\frac{p_{k,j}}{b_k}),$$

where $M_{k,j}$ is the element score and $p_{k,j}$ is the probability of observing the amino acid k at the ANARCI-numbered position j in the cluster, and b_k is the background probability of amino acid k, which is considered to be the same for all amino acid types (*i.e.* 0.05) A pseudo-count of 0.001 was added to all elements with no observations to prevent computational errors.

To make a cluster prediction, we only considered clusters that contain members of the same sequence length as the target sequence. The PSSM score for a target sequence, s_c , for cluster c is:

$$s_c = \sum_{j=J_0}^{J} M_{k,j}$$

where J is the set of positions in the target sequence. If the maximum total score is above an assignment threshold (see S3), an assignment is made to the cluster with the maximum total score.

In our dataset, we observed that 99.3% (2721/2741) length-8 L2 loops are clustered in the L2-8-A. Henceforth, we decided to alter the assignment method: all L2 loops of the dominant length (length-8 according to North *et al.*, 2011) were assigned to a single cluster; the remaining loops were not assigned to any clusters. This resulted in the same precision and recall as the selected threshold (-1; see S3).

S3 Cross-validation for threshold selection

We carried out leave-one-out cross-validation on the unique CDR sequences of the SAbDab set. Within a cluster, only unique sequences were retained. For non-clustered sequences, only unique sequences in the set were retained.

For each loop, if the backbone RMSD between the actual structure and any members of the assigned cluster is <1.5 Å, this was labelled a **true positive (TP)**; otherwise this was labelled a **false positive (FP)**. If the loop was not in any cluster and was not assigned to any cluster, this was labelled a **true negative (TN)**. If the loop was in a cluster but was not assigned to any cluster, this was labelled a **false negative (FN)**.

We used the following definitions for the calculation of recall, precision and coverage:

- Recall = $\frac{TP}{TP+FN}$
- Precision = $\frac{TP}{TP+FP}$
- Coverage = $\frac{TP+FP}{TP+TN+FP+FN}$

For each CDR, we calculated the precision and recall for different assignment thresholds. We then calculated the F_1 score for each threshold:

$$F_\beta = (1+\beta^2)\,\frac{precision \cdot recall}{(\beta^2 \cdot precision) + recall} \text{ where } \beta = 1$$

The maximum total scores were between -4 and 4 (Figure S3.1), hence we performed a parameter sweep over threshold values using an increment of 0.5.

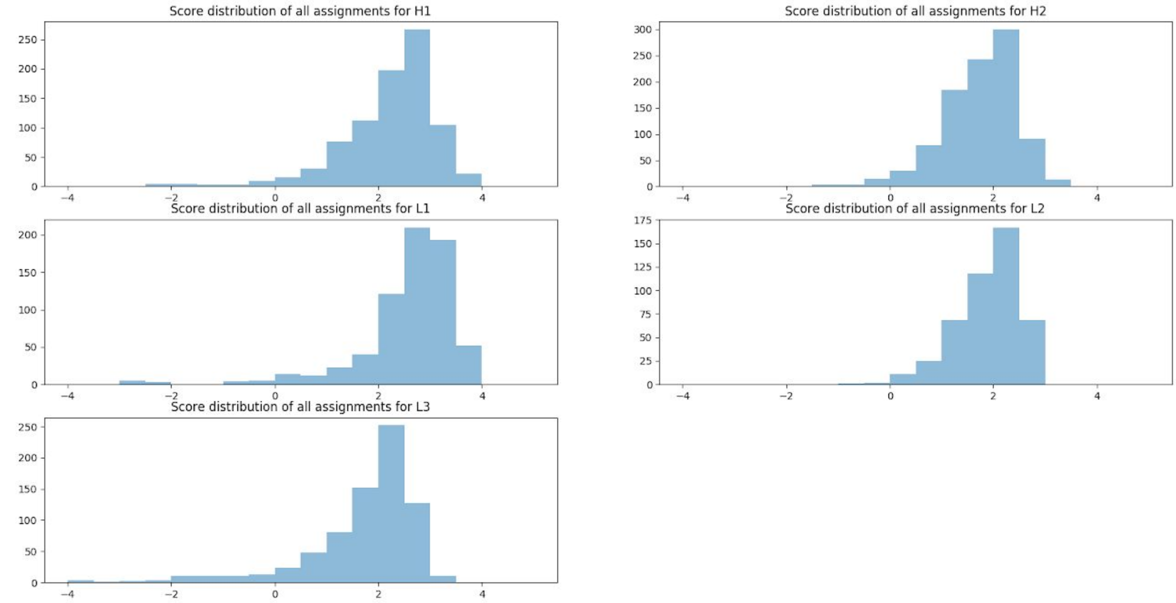

Figure S3.1 Maximum total score distributions for different CDR loops during cross-validation.

We selected the scoring threshold with the highest F_1 score (Table S3.1). Table S3.2 shows the corresponding recall, precision and coverage of the selected threshold for each CDR type.

Table S3.1 F_1 score from the cross-validation. The highlighted cells indicate the maximum F1-score across the different thresholds for each CDR.

Assignment Thresholds	H1	H2	L1	L2	L3
-2	0.9337	0.9661	0.9765	0.9956	0.9541
-1.5	0.934	0.9661	0.9765	0.9956	0.9601
-1	0.9338	0.966	0.9765	0.9956	0.9619
-0.5	0.9342	0.9664	0.9771	0.9945	0.9615
0	0.9363	0.9639	0.9755	0.9923	0.961
0.5	0.9408	0.9526	0.9736	0.9811	0.9571
1	0.9333	0.9259	0.9663	0.9521	0.9347
1.5	0.9135	0.8324	0.9537	0.8649	0.8818

Table S3.2 Recall, precision, accuracy and coverage at the selected thresholds.

CDR (threshold)	Recall (%)	Precision (%)	Accuracy (%)	Coverage (%)
H1 (0.5)	99.45	89.26	89.47	93.75
H2 (-0.5)	99.89	93.6	93.65	97.54
L1 (-0.5)	99.84	95.67	95.64	97.38
L2 (-1)	100	99.13	99.14	98.5
L3 (-1)	99.26	93.31	93.22	91.69

S4 Blind test set for SCALOP prediction performance

We collected all redundant structures that fulfil the quality requirements of the SCALOP database and became available in SAbDab (Dunbar *et al.*, 2014) between 1st August 2017 and 31st May 2018. We used SCALOP to predict the canonical forms of this blind test set of CDR loops using the database constructed with structures available before 1st July 2017. The definition of the performance indicators (TP, TN, FP and FN) are the same as in S3. The performance is shown in Table S4.1. SCALOP maintains high coverage and precision on the new structures.

Table S4.1 Coverage and precision of the leave-one-out cross-validation on SCALOP's model selection.

	**H1**	**H2**	**L1**	**L2**	**L3**
**Coverage**	94.17%	95.12%	96.76%	97.38%	89.60%
**Precision**	88.79%	87.13%	94.53%	99.22%	91.33%

A target structure with a root-mean-square deviation of less than 1.5 Å to the predicted structure is considered correct.

S5 Cross-validation for structure selection

SCALOP can generate a model structure if the user supplies a structure of the framework.

After SCALOP assigned a canonical form to the input sequence, a structural prediction is selected from the members of the assigned cluster. We calculate the Environment Substitution Score (ESS; from FREAD, Krawczyk *et al.*, 2018) of the members and the target sequence. Among the member structures with an ESS of >25, we calculated their anchor RMSD with the input structure. Two residues before and after the member CDR loop structures were used as the anchor to compute the anchor RMSD of their backbone atoms. We then select the member with the highest ESS and lowest anchor RMSD as the structural prediction.

We carried out a leave-one-out cross-validation study on the structural prediction. We used the unique CDR sequences of the SAbDab set described in the Supplementary Materials, left one sequence out each time when constructing the PSSM, and predicted the canonical form of the target sequence. From the members of the assigned cluster, the CDR structure with the highest ESS and lowest anchor RMSD with the target sequence's native framework structure is selected, barring all sequence-identical structures. If the selected structure has a backbone RMSD of less than 1.5 Å with the native structures of any of the sequence-identical loops, the prediction is considered a **true positive (TP)**; otherwise, it is a **false postive (FP)**. If no structures were assigned and no clustered structures fall within 1.5 Å backbone RMSD from the native structures of any of the sequence-identical loops, this is considered a **true negative (TN)**; otherwise it is a **false negative (FN)**.

This results in the prediction performance shown in Table S5.1. Using the same dataset as in the paper, the results are similar to those in Table 1 in the main text. H1 and H2 see a drop in precision as this simple method does not always select the best CDR structure from the large clusters seen for H1 and H2.

Table S5.1 Coverage and precision of the leave-one-out cross-validation on SCALOP's model selection.

	**H1**	**H2**	**L1**	**L2**	**L3**
**Coverage**	93.75%	97.03%	97.38%	98.50%	89.26%
**Precision**	81.73%	80.23%	92.68%	98.04%	86.53%

A target structure with a root-mean-square deviation of less than 1.5 Å to the predicted structure is considered correct.

S6 Benchmark with FREAD

We ran FREAD (Deane and Blundell, 2001; Choi and Deane, 2010; Krawczyk *et al.*, 2018) using only structures whose PDB code and chain identifier are found in the SCALOP database. To select a prediction, we used length-dependent environment substitution score (ESS) cut-offs:

- Lengths < 13: 25
- Lengths 13-16: 40
- Lengths > 16: 55

The decoy with the top ESS score above the length-dependent ESS cut-off, and the lowest anchor RMSD with the model framework was selected as the FREAD prediction. FREAD does not make a prediction if none of the decoys are above the corresponding ESS cut-off, or when the decoy has an anchor RMSD of ≥ 1 Å. No structural models are generated from FREAD. We only took the PDB code and chain identifier of the selected decoy structure.

S6.1 Coverage and precision of FREAD on loops in the SCALOP database

We ran a leave-one-out cross-validation on all structures within the SCALOP database. For each case, the frameworks and CDR loops from identical antibody sequences were eliminated. The same measures of correctness were used for FREAD as for SCALOP. A **true positive** prediction refers to a case where the backbone RMSD between the actual and predicted structures was < 1.5 Å; otherwise it was a **false positive**. If the minimal backbone RMSD between the actual structure and any loop structures was ≥ 1.5 Å, it was considered a **true negative** if FREAD does not make a prediction; otherwise the lack of a FREAD prediction was considered a **false negative**. The calculation for the coverage and precision are the same as in S3. Table S6.1 shows the results of the cross-validation.

Table S6.1 Precision and prediction coverage of FREAD on loops in the SCALOP database

	**Precision**	**Coverage**
**H1**	80.19%	96.79%
**H2**	88.50%	93.38%
**L1**	92.72%	98.76%
**L2**	98.27%	98.89%
**L3**	91.29%	98.02%

S7 Performance of SCALOP and FREAD on next generation sequencing (NGS) data

We ran SCALOP and FREAD on a set of ~8 million light chain and ~5 million heavy chain sequences (Krawczyk *et al.*, 2018; referred to as 'NGS data'), and compared their predictions to assess overlap coverage, consistency and speed.

The "overlap coverage" is the percentage of sequences for which both FREAD and SCALOP made a prediction. Within the overlapped predictions, if the FREAD prediction was < 1.5 Å backbone RMSD from any member of the cluster assigned by SCALOP, it was considered a consistent prediciton.

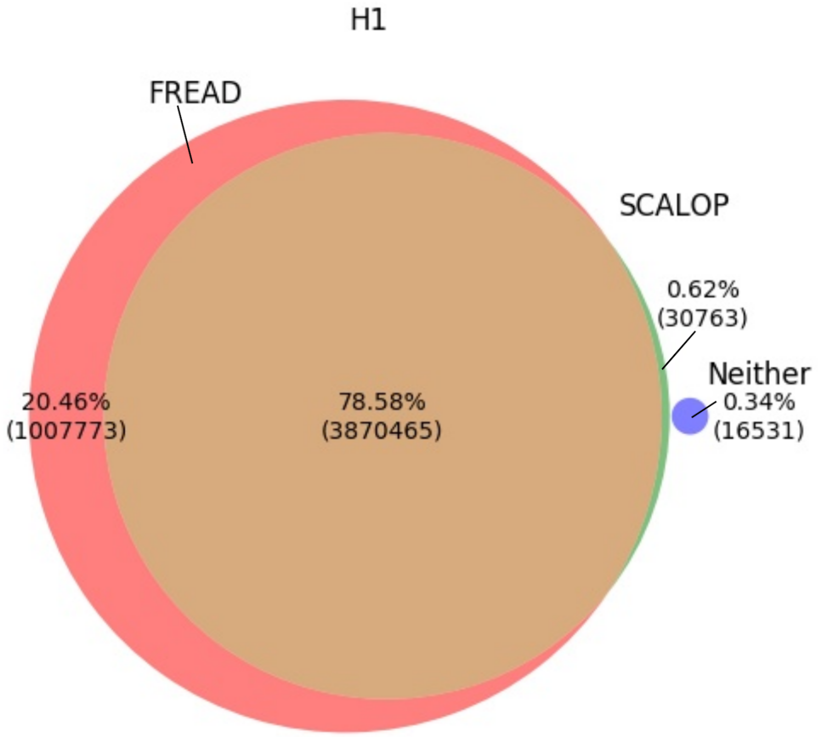

Figure S7.1 Prediction coverage of SCALOP and FREAD on the NGS data, for H1 loops. FREAD has higher coverage than SCALOP, but the overlap coverage is close to 80%. Only 0.34% of sequences were not predicted by either FREAD nor SCALOP.

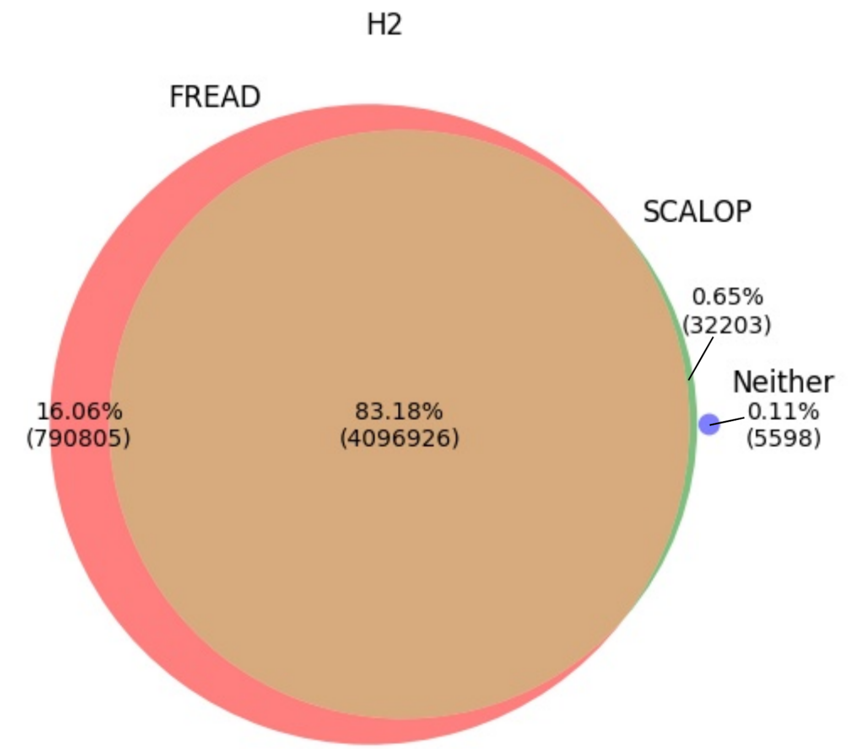

Figure S7.2 Prediction coverage of SCALOP and FREAD on the NGS data, for H2 loops. FREAD has higher coverage than SCALOP and the overlap coverage is above 80%. Only 0.11% of all sequence data is predicted by neither method.

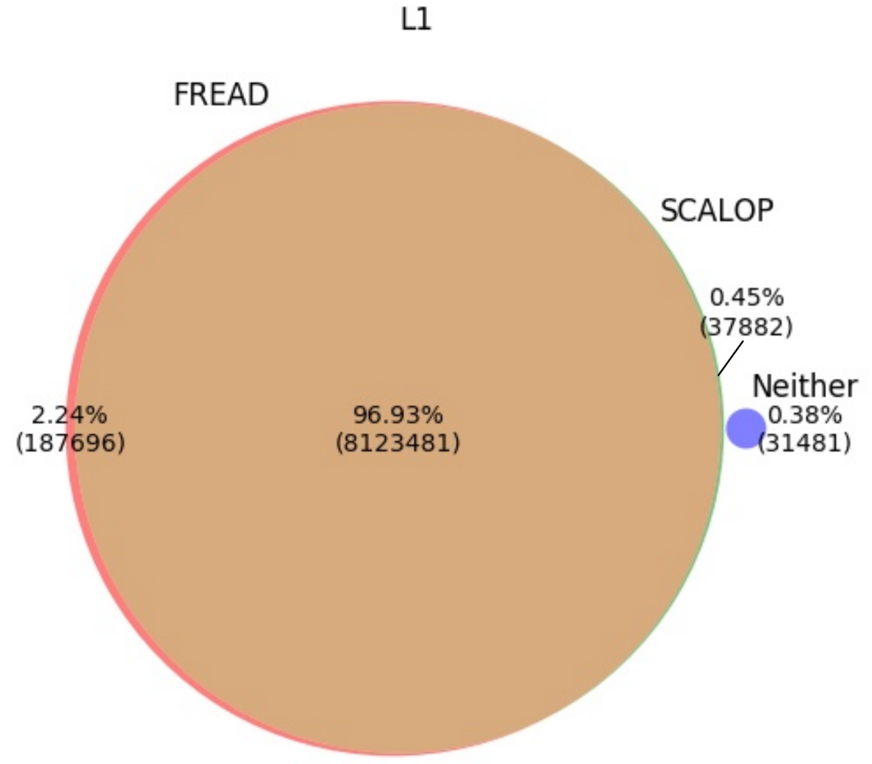

Figure S7.3 Prediction coverage of SCALOP and FREAD on the NGS data, for L1 loops. The coverage of SCALOP and FREAD are comparable while the overlapping coverage is close to 97%.

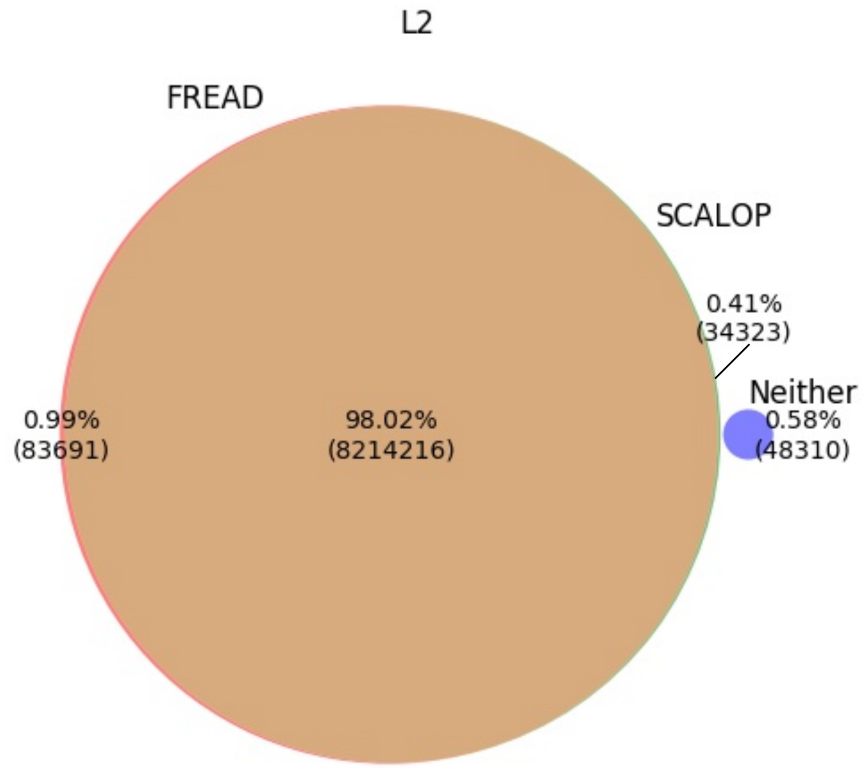

Figure S7.4 Prediction coverage of SCALOP and FREAD on the NGS data, for L2 loops. SCALOP and FREAD make predictions over almost the same set of loops.

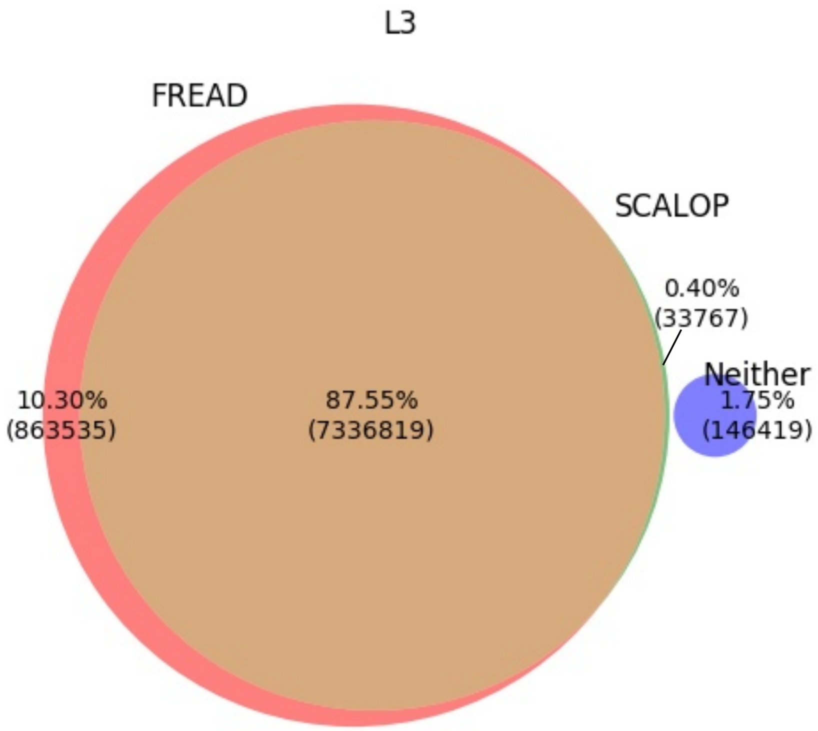

Figure S7.5 Prediction coverage of SCALOP and FREAD on the NGS data, for L3 loops. FREAD has a higher coverage than L3, but the overlap coverage is reasonably high for a loop that is marginally more variable than the other CDRs with canonical forms.

Table S7.1 Overlap coverage and consistent prediction within the overlap in NGS set

CDR	Overlap Coverage (%)	Consistent prediction (% of overlap coverage)
H1	78.58	95.15
H2	83.18	95.24
L1	96.93	100
L2	98.02	100
L3	87.55	100

S8 Backdating the SCALOP database: Performance evaluation

We used the database of loop structures available as of 1st July, 2018 as the test set. For the back-dated set, we selected loops whose deposition dates were before the end of the year of interest. We chose the representative years based on the publication dates of previous canonical forms definitions (Al-Lazikani *et al.*, 1997; North *et al.*, 2011; Nowak *et al.*, 2016) and the most recent year (2017). For each back-dated set, we carried out a leave-one-out cross-validation for all the loops in the test set:

- for loops that existed on or before the given year, we did not include the loop of interest in the construction of PSSMs, and
- for loops that came into existence later, we built the PSSMs based on all loops present in the back-dated set.

The results show that by updating the database, prediction coverage increases while retaining high precision.

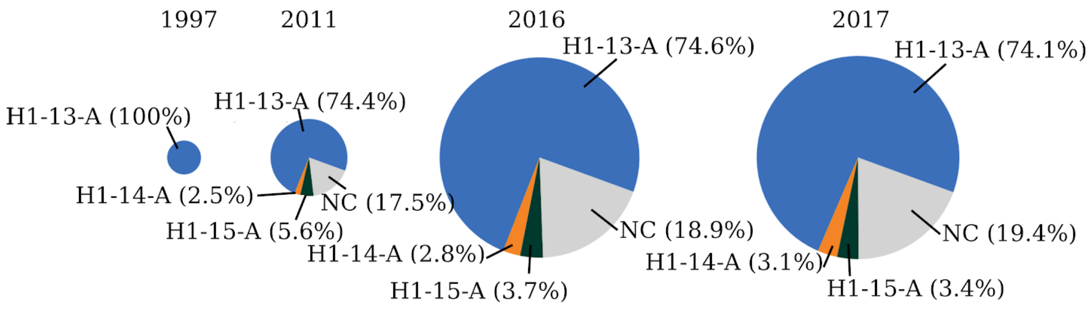

	1997	2011	2016	2017
# Sequences	146	1467	2695	3022
Coverage (%)	78.6	91.6	92.5	92.5
Precision (%)	90.7	90.4	90.5	90.5

Figure S8.1 The changes in H1 cluster composition and their prediction coverage and precision. The radii of the pie charts are proportional to the log(#Sequences). NC refers to non-clustered sequences. The number of H1 sequences increased by 20-fold within 20 years. Length-14 and length-15 clusters were absent in 1997, but appear from 2011.

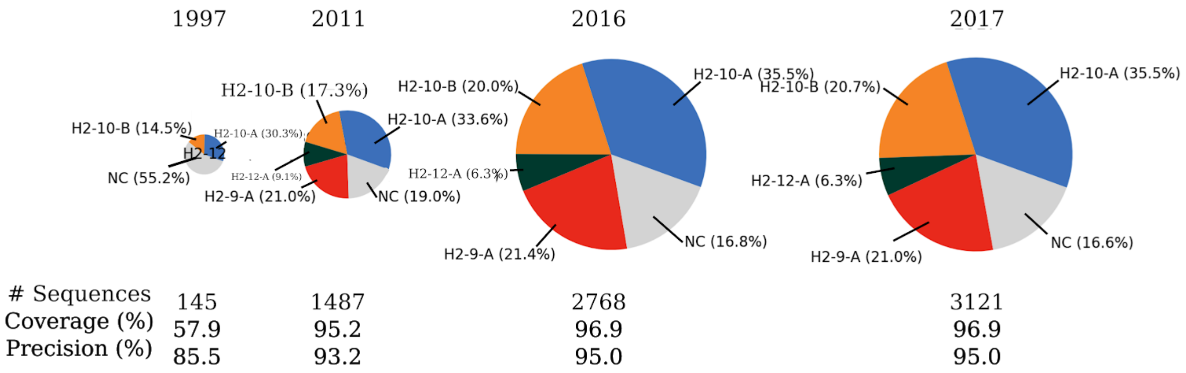

Figure S8.2 The changes in H2 cluster composition and their prediction coverage and precision. The radii of the pie charts are proportional to the *log*(#Sequences). NC refers to non-clustered sequences. A near 40% increase of prediction coverage is seen over 20 years. The portion of non-clustered sequences (16.8%) has dropped to one-third of the portion in 1997 (55.2%).

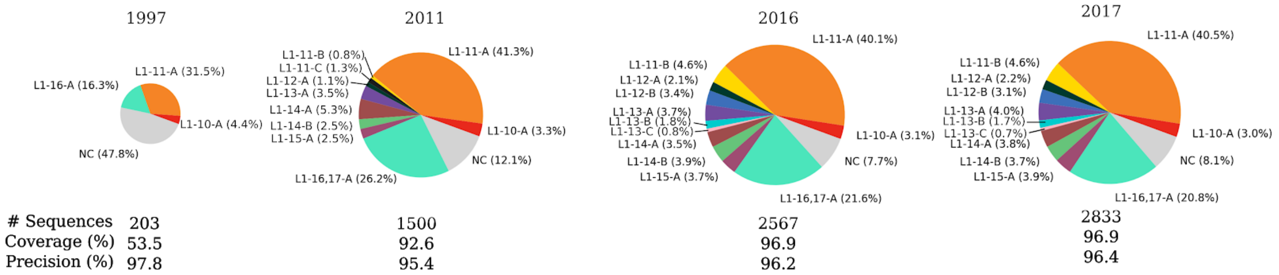

Figure S8.3 The changes in L1 cluster composition and their prediction coverage and precision. The radii of the pie charts are proportional to the *log*(#Sequences). NC refers to non-clustered sequences. The number of clusters grew from 4 in 1997 to 12 in 2016. Length-17 sequences joined the length-16 cluster in 1997 to form the L1-16,17-A cluster. The 2011-L1-11-C cluster combined with the L1-11-B cluster and resulted in the 2016-L1-11-B cluster. The portion of non-clustered sequences in 2016 (7.7%) dropped to a quarter of the portion in 1997 (47.8%).

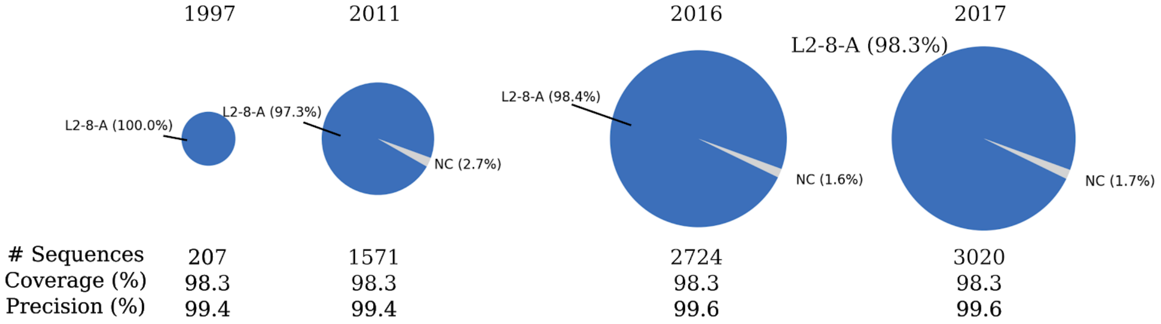

Figure S8.4 The changes in L2 cluster composition and their prediction coverage and precision. The radii of the pie charts are proportional to the *log*(#Sequences). NC refers to non-clustered sequences. The conformation of L2 loop is largely invariant, hence they only belong to one cluster.

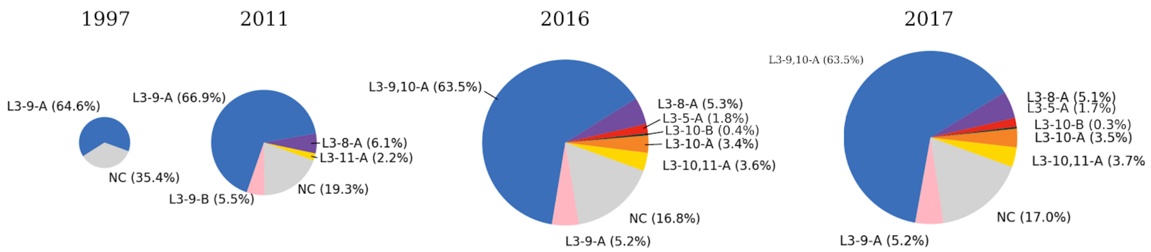

# Sequences	206	1532	2672	2955
Coverage (%)	61.1	77.8	91.0	91.1
Precision (%)	97.4	96.2	94.0	94.0

Figure S8.5 The changes in L3 cluster composition and their prediction coverage and precision. The radii of the pie charts are proportional to the *log*(#Sequences). NC refers to non-clustered sequences. In 1997, only one length-9 cluster existed. Between 2011-2016, some length-10 sequences joined the 2011-L3-9-A cluster, which becomes the 2016-L3-9,10-A cluster. Likewise, some length-10 loops joined the 2011-L3-11-A cluster to form 2016-L3-10,11-A.

References

Al-Lazikani B., Lesk A.M., Chothia C. (1997) Standard conformations for the canonical structures of immunoglobulins. *J. Mol. Biol.,* **273**, 927–948.

Choi, Y. and Deane, C. M. (2010). FREAD revisited: Accurate loop structure prediction using a database search algorithm. *Proteins*, **78**(6), 1431–1440.

Deane, C. M. and Blundell, T. L. (2001). CODA: a combined algorithm for predicting the structurally variable regions of protein models. *Protein Sci.*, **10**(3), 599–612.

Dunbar, J., Krawczyk, K., Leem, J., Baker, T., Fuchs, A., Georges, G., Shi, J., and Deane, C. M. (2014). SAbDab: the structural antibody database. *Nucleic Acids Res.*, **42**(D1), D1140–D1146.

Lefranc, M. P., Giudicelli, V., Ginestoux, C., Jabado-Michaloud, J., Folch, G., Bellahcene, F., Wu, Y., Gemrot, E., Brochet, X., Lane, J., Regnier, L., Ehrenmann, F., Lefranc, G., and Duroux, P. (2009). IMGT®, the international ImMunoGeneTics information system®. *Nucleic Acids Res.*, **37**(Suppl. 1), D1006–12.

Krawczyk, K., Kelm, S., Kovaltsuk, A., Galson, J. D., Kelly, D., Trück, J., Regep, C., Leem, J., Wong, W. K., Nowak, J., Snowden, J., Wright, M., Starkie, L., Scott-Tucker, A., Shi, J. and Deane, C. M. (2018). Structural Mapping Antibody Repertoires. *Front. Immunol.* DOI:10.3389/FIMMU.2018.01698.

North, B., Lehmann, A., and Dunbrack, R. L. (2011). A new clustering of antibody CDR loop conformations. *J. Mol. Biol.*, **406**(2), 228–256.

Nowak, J., Baker, T., Georges, G., Kelm, S., Klostermann, S., Shi, J., Sridharan, S., and Deane, C. M. (2016). Length-independent structural similarities enrich the antibody CDR canonical class model. *mAbs*, **8**(4), 751–760.
